# Acute alcohol administration dampens central extended amygdala reactivity

**DOI:** 10.1038/s41598-018-34987-3

**Published:** 2018-11-12

**Authors:** Juyoen Hur, Claire M. Kaplan, Jason F. Smith, Daniel E. Bradford, Andrew S. Fox, John J. Curtin, Alexander J. Shackman

**Affiliations:** 1 Department of Psychology, University of Maryland, College Park, MD 20742 USA; 2 Neuroscience and Cognitive Science Program, University of Maryland, College Park, MD 20742 USA; 3 Maryland Neuroimaging Center, University of Maryland, College Park, MD 20742 USA; 4 Department of Psychology, University of Wisconsin—Madison, 1202 West Johnson Street, Madison, WI 53706 USA; 5 Department of Psychology, University of California, Davis, CA 95616 USA; 6 California National Primate Research Center, University of California, Davis, CA 95616 USA

## Abstract

Alcohol use is common, imposes a staggering burden on public health, and often resists treatment. The central extended amygdala (EAc)—including the bed nucleus of the stria terminalis (BST) and the central nucleus of the amygdala (Ce)—plays a key role in prominent neuroscientific models of alcohol drinking, but the relevance of these regions to acute alcohol consumption in humans remains poorly understood. Using a single-blind, randomized-groups design, multiband fMRI data were acquired from 49 social drinkers while they performed a well-established emotional faces paradigm after consuming either alcohol or placebo. Relative to placebo, alcohol significantly dampened reactivity to emotional faces in the BST. To rigorously assess potential regional differences in activation, data were extracted from unbiased, anatomically predefined regions of interest. Analyses revealed similar levels of dampening in the BST and Ce. In short, alcohol transiently reduces reactivity to emotional faces and it does so similarly across the two major divisions of the human EAc. These observations reinforce the translational relevance of addiction models derived from preclinical work in rodents and provide new insights into the neural systems most relevant to the consumption of alcohol and to the initial development of alcohol abuse in humans.

## Introduction

Alcohol use is common (nearly three-quarters of Americans consumed some form of ethanol in the past year and, among them, 17.5% met criteria for an alcohol use disorder), contributes to a variety of adverse outcomes, and imposes a rapidly growing burden on public health and the economy^1–3^, highlighting the need to understand the acute impact of alcohol consumption on the human brain.

**Figure 1:**
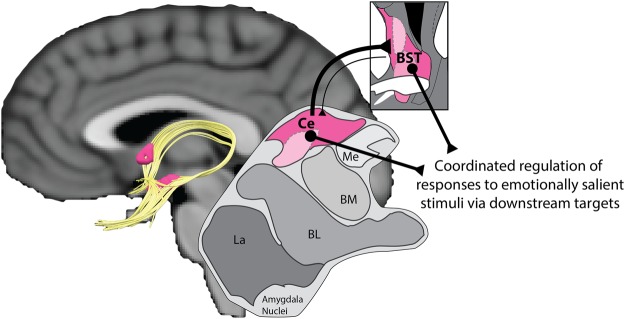
Human EAc. The EAc (*magenta*) encompasses the BST (encircling the anterior commissure) and the Ce (within the dorsal portion of the amygdala proper). The BST and the Ce are anatomically interconnected via the *ventral amygdalofugal pathway* and the *stria terminalis*, as indicated by deterministic tractography (*gold*). Both regions are poised to orchestrate responses to emotionally salient stimuli via dense projections to downstream effector regions. Portions of this figure were adapted from ref.^71^. Abbreviations—BL, basolateral nucleus of the amygdala; BM, basomedial nucleus of the amygdala; BST, bed nucleus of the stria terminalis; Ce, central nucleus of the amygdala; La, lateral nucleus of the amygdala; Me, medial nucleus of the amygdala.

Evidence gleaned from animal models highlights the potential importance of the central extended amygdala (EAc), including the bed nucleus of the stria terminalis (BST) and the central nucleus of the amygdala (Ce)^4,5^ (Fig. 1). The BST and the Ce show similar patterns of connectivity, cellular composition, neurochemistry, and gene expression, and both are critical for triggering defensive responses to threat^4,6–9^. Through dense projections to downstream effector regions, these regions play an important role in prioritizing the processing of salient social cues, such as facial expressions of emotion^10,11^, and shaping social interactions^12,13^. The EAc also plays a key role in prominent neuroscientific models of alcohol-drinking^14–18^, with work in rodents indicating that alcohol acutely dampens EAc reactivity^19–23^.

The acute impact of alcohol on BST or Ce function in humans remains unclear. To date, imaging research has focused on the role of the amygdala proper, with several small-scale studies reporting evidence suggestive of dampened reactivity to emotional faces, particularly those expressing fear or anger (Table 1)^24–27^. None directly examined either the BST or the Ce, despite their central role in prominent models of alcohol consumption. Here, we used a novel combination of approaches to rigorously assess the impact of acute alcohol consumption on EAc reactivity for the first time. Using a single-blind, randomized-groups design and ecologically relevant dosing (Table 2), fMRI data were acquired from 49 psychiatrically healthy social drinkers while they performed an fMRI-optimized emotional-faces/places paradigm after consuming alcohol or placebo. The choice of paradigm was rooted in work demonstrating that the amygdala is robustly activated by emotional faces, particularly those depicting expressions of fear^28–31^. This has motivated the use of similar paradigms in work focused on the development of anxiety and mood disorders^32,33^ and the acute impact of alcohol and pharmaceutical (e.g. benzodiazepine) interventions^24–27,34,35^, as well as large-scale neuroimaging initiatives (e.g., Human Connectome Project, UK BioBank)^36,37^. Several methods served to enhance neuroanatomical resolution, including a multiband pulse sequence and advanced co-registration and spatial normalization techniques^38^ (Table 2). Recently developed, anatomically defined regions-of-interest (ROIs)^39,40^ made it possible to directly compare the hypothesized dampening effects of alcohol in the BST and the Ce in an unbiased manner. Understanding the acute consequences of alcohol for EAc function is important. It would clarify whether models of substance abuse derived from work in rodents—a species that diverged from the ancestors of modern humans ~75 million years ago^41^—are relevant to human alcohol consumption^14,15^. It also promises to inform our understanding of work linking variation in EAc function to the emergence of alcohol abuse^42,43^ and to provide insight into the EAc’s role in recreational drinking.

**Table 1:** The effects of acute alcohol administration on amygdala reactivity in human imaging studies.

Study	*N* (% Male)	EPI Voxel Size (mm^3^)	Normalization^a^	Design	Task	Amygdala Results
Present study	49 (53%)	8.0	FSL (BBR) and ANTS (diffeomorphic)	Single-blind, placebo-controlled, randomized groups	Fearful/Neutral Faces vs. Places (blocked)	See the main report
Gilman 2008	12 (42%)	70.3	“AFNI” (affine?)	Double-blind, placebo-controlled, randomized cross-over	Fearful vs. Neutral Faces (event-related)	***Voxelwise*** *a*. *Expression* × *Treatment*, *NS* *b*. *Expression* during Placebo: Fearful > Neutral, *p* < 0.05, corrected *c*. *Expression* during Alcohol: Fearful vs. Neutral, *NS* ***ROI*** *a*. *Expression* × *Treatment*, *p* = 0.08 *b*. *Expression* during Placebo: Fearful > Neutral, *p* < 0.05 c. *Expression* during Alcohol: Fearful vs. Neutral, *NS*
Gilman 2012^b^	14 (100%)	70.3	“AFNI” (affine?)	Double-blind, placebo-controlled, randomized cross-over	Fearful vs. Neutral Faces (event-related)	***Voxelwise*** *a*. *Expression* × *Treatment*, *NR* *b*. *Expression* during Placebo: Fearful > Neutral, *p* < 0.01, corrected *c*. *Expression* during Alcohol: Fearful vs. Neutral, *NS* ***ROI*** *a*. *Expression* × *Treatment*, *p* = 0.02 *b*. *Treatment* for Neutral: *NR* *c*. *Treatment* for Fearful: Alcohol < Placebo, *p* = 0.02
Padula 2011^c^	12 (58%)	58.8	“AFNI” (affine?)	Single-blind, placebo-controlled, randomized cross-over	Angry/Fearful/Happy Faces vs. Shapes (blocked)	***Voxelwise*** *a*. *Stimulus* × *Treatment*, *NS* *b*. *Stimulus* during Placebo: Faces > Shapes, *p* < 0.05, corrected *c*. *Stimulus* during Alcohol: Faces vs. Shapes, *NS*
Sripada 2011	12 (83%)	70.3	SPM12 (EPI template)	Double-blind, placebo-controlled, randomized cross-over	Fearful/Angry vs. Happy Faces (blocked)	***Voxelwise*** *a*. *Expression* × *Treatment*, *NS* *b*. *Expression* during Placebo: Fearful/Angry > Happy, *p* < 0.005, uncorrected *c*. *Expression* during Alcohol: Fearful/Angry vs. Happy, *NS* *d*. *Treatment* during Fearful/Angry: Alcohol < Placebo, *p* < 0.005, uncorrected *d*. *Treatment* during Happy: *NR* ***ROI*** *a*. *Expression* × *Treatment*, *NR* *b*. *Expression* during Placebo: Fearful/Angry > Happy, *p* < 0.05 *c*. *Expression* during Alcohol: Alcohol < Placebo, *NS*

^a^Older normalization techniques (e.g., affine, EPI-to-EPI) can introduce substantial spatial smoothing and registration error, which is a concern for work focused on small subcortical structures, such as the EAc. ^b^Social drinker (‘control’) group. ^c^ROI analyses were not reported. Abbreviations—BBR, boundary-based registration of the T1- and T2-weighted images; *NR*, not reported; *NS*, not significant.

**Table 2:** Demographic variables and descriptive statistics for the placebo and alcohol groups.

	Total	Placebo	Alcohol	Difference
Sample size	49	22	27	N/A
Mean Age in Years (*SD*)	22.4 (2.5)	22.1 (1.4)	22.6 (3.1)	*t*(47) = 0.69, *p* = 0.50
Gender: Female/Male	23/26	11/11	12/15	χ^2^ = 0.15, *p* = 0.8
Mean BAL^a^ (*SD*)	N/A	0.00 (0.00)	0.09 (0.02)	*t*(47) = 27.20, *p* < 0.001
Mean Subjective Estimate of Number of Drinks Consumed During the Study (*SD*)	N/A	2.07 (1.09)^b^	4.56 (1.25)^c^	*t*(47) = 7.32, *p* < 0.001
Mean Motion, Frame-to-Frame Displacement (*SD*)	0.13 (0.03)	0.12 (0.03)	0.13 (0.03)	*t*(47) = 1.01, *p* = 0.32

^a^Pre-MRI and post-MRI BAL were strongly correlated, *r*(47) = 0.96, *p* < 0.001. ^b^Within-group difference from zero, *t*(21) = 8.87, *p* < 0.001. ^c^Within-group difference from zero, *t*(26) = 18.93, *p* < 0.001.

## Method

### Subjects

A total of 61 individuals between the ages of 21 and 35 years were recruited from the community as part of a larger study. All had experience with the highest study dose of alcohol used in the present study (~4–5 standard drinks) within the past 12 months, normal or corrected-to-normal color vision, and reported the absence of lifetime alcohol or substance-related problems, lifetime neurological symptoms, current psychiatric diagnosis or treatment, pervasive developmental disorder or very premature birth, or a medical condition that would contraindicate either acute alcohol consumption or MRI. Twelve subjects were excluded from analyses due to unusable T1-weighted datasets (*n* = 3), technical problems with the scanner (*n* = 1), incidental neurological findings (*n* = 2), inadequate behavioral performance (>2 *SD*s below the mean; *n* = 3), or excessive motion artifact (*n* = 3; see below), yielding a final sample of 49 subjects (46.9% female; Table 2 in the main report). All procedures were approved by the University of Maryland Institutional Review Board and carried out in accordance with the relevant guidelines and regulations. Subjects provided informed written consent.

### Overview and General Procedures

Subjects abstained from alcohol and other substances for 24 hours and food/drink for 3 hours prior to the session. At the start of the session, initial sobriety was confirmed using a standard breath assay (Alcosensor IV Breathalyzer; Intoximeters Inc., St. Louis, MO). Subjects were randomly assigned (stratified by sex and race/ethnicity) to receive an alcoholic or placebo beverage, which was consumed just prior to scanning. The decision to employ a between-subjects design was motivated by work underscoring the relatively low test-retest stability of fMRI measures of amygdala reactivity, which renders it suboptimal for randomized cross-over designs^44,45^. Blood alcohol level (BAL) was assessed immediately before and after scanning. Subject status was continuously monitored using an MRI-compatible eye-tracker. At the end of the session, subjects estimated the number of standard alcoholic drinks that they had consumed.

#### Alcohol/Placebo Procedures

Well-established procedures were used for administering alcohol or placebo^46^. Consistent dosing was achieved using a formula that uses height, weight, age, and sex to produce the target BAL of ~0.09% ~30 minutes after the completion of beverage consumption (range: 0.06–0.12%; Table 2)^47,48^. Alcoholic beverages contained a mixture of juice and 100-proof vodka. To control absorption, subjects consumed 3 equal doses over 30 minutes. The placebo group received a similar beverage, with distilled water replacing the vodka. Subjects assigned to the alcohol (or placebo) group observed the experimenter pouring the vodka (or distilled water) from a vodka bottle. The placebo manipulation was reinforced by floating 3 ml of bitters and 3 ml of vodka on the surface of the beverage and delivering a minute amount of aerosolized vodka to the rim of the beverage containers outside the subject’s view. Immediately following consumption of the third beverage, BAL was assessed and subjects were scanned. BAL was re-assessed immediately following the final scan (inter-assessment period: *M* = 70 min, *SD* = 6.0 min), as in prior work^49^. On average, subjects in the placebo group estimated that they consumed ~2 drinks, confirming the efficacy of the placebo manipulation (Table 2).

#### Emotional-Faces/Places Paradigm

To assess the impact of acute alcohol administration on EAc function, imaging data were acquired while subjects performed a simple, fMRI-optimized, continuous-performance task. Building on work by our group^38,50^ and many others^24–35,51^ demonstrating the utility of emotional face paradigms for probing amygdala reactivity—particularly when compared to low-level perceptual control stimuli—subjects viewed alternating blocks of either emotional faces (8 blocks) or places (9 blocks). The use of a block design enhances detection power and mitigates potential concerns about alcohol-induced changes in the shape of the hemodynamic response function (HRF)^52,53^. Block length (~16.3 s) was also optimized to detect differential blood oxygen level-dependent (BOLD) signals across the two conditions^52,53^. To maximize signal strength and homogeneity and minimize potential neural habituation^52–54^, each block consisted of 16 brief presentations of faces or places (~1.02 s/image). During face blocks, subjects discriminated (two-alternative/forced-choice) between fearful (75% trials) and emotionally neutral facial expressions (25% trials) presented in a pseudorandomized order. This design choice was aimed at reducing monotony and minimizing potential habituation of the amygdala^54^. Face stimuli were adapted from prior work by Gamer and colleagues^55,56^ and included standardized images of unfamiliar male and female adults displaying unambiguous fearful or neutral expressions. To maximize the number of models and mitigate potential habituation, images were derived from several well-established databases: Ekman and Friesen’s Pictures of Facial Affect^57^, the FACES database^58^, the Karolinska Directed Emotional Faces database (http://www.emotionlab.se/resources/kdef), and the NimStim Face Stimulus Set (https://www.macbrain.org/resources.htm). Color images were converted to grayscale, brightness normalized, and masked to occlude non-facial features (e.g., ears, hair). During place blocks, subjects discriminated between suburban residential buildings (i.e. houses; 75%) and urban commercial buildings (i.e. skyscrapers; 25%). Grayscale place stimuli were adapted from prior work^59,60^. Responses were made using an MRI-compatible, fiber-optic response pad (MRA, Washington, PA).

#### MRI Data Acquisition

MRI data were acquired using a Siemens Magnetom TIM Trio 3 Tesla scanner and 32-channel head-coil. Sagittal T1-weighted anatomical images were acquired using a MPRAGE sequence (TR = 1,900 ms; TE = 2.32 ms; inversion time = 900 ms; flip angle = 9°; sagittal slice thickness = 0.9 mm; in-plane = 0.449 × 0.449 mm; matrix = 512 × 512; field-of-view = 230 × 230). To enhance resolution, a multi-band sequence was used to collect a total of 286 oblique-axial echo planar imaging (EPI) volumes during the faces/places task (multiband acceleration = 6; TR = 1,000 ms; TE = 39.4 ms; flip angle = 36.4°; slice thickness = 2.2 mm, number of slices = 60; in-plane resolution = 2.1875 × 2.1875 mm; matrix = 96 × 96). Images were collected in the oblique axial plane (approximately −20° relative to the AC-PC plane) to minimize susceptibility artifacts. To enable fieldmap correction, two oblique-axial spin echo (SE) images were collected in each of two opposing phase-encoding directions (rostral-to-caudal and caudal-to-rostral) at the same location and resolution as the functional volumes (i.e., co-planar; TR = 7,220 ms; TE = 73 ms).

#### MRI Data Preprocessing

Given our focus on the EAc, methods were optimized to minimize spatial normalization error and other potential sources of noise. All MRI data were visually inspected before and after processing for quality assurance purposes.

Anatomical Data Processing: Methods are similar to those described in other recent reports by our group^38,40^. T1 images were brain-extracted (‘skull-stripped’) using a multi-tool approach^40^. Brain-extracted T1 images were normalized to the MNI152 template using the high-precision diffeomorphic approach implemented in *SyN*^61^. The mean of the normalized T1 images is depicted in Supplementary Figure S1. FSL was used to create a fieldmap and undistorted SE image.

Functional Data Processing: The first 3 volumes of each EPI scan were removed. Remaining volumes were de-spiked and slice-time corrected using AFNI^62^. For co-registration of the functional and anatomical images, an average EPI image was created. The average image was simultaneously co-registered with the corresponding T1-weighted image in native space and corrected for geometric distortions using the boundary-based registration method implemented in FSL and the previously created fieldmap, undistorted SE image, and T1 image. Spatial transformations were concatenated and applied to the functional data in a single step. The transformed images were re-sliced (2-mm^3^), smoothed (6-mm), and filtered (0.0078125-Hz high-pass). To assess residual motion artifact, the variance of volume-to-volume displacement of a selected voxel in the center of the brain (*x* = 5, *y* = 34, *z* = 28) was calculated using the motion-corrected EPI data. Subjects (*n* = 3) with extreme motion variance (>2*SD*s above the mean) were excluded from analyses.

fMRI Modeling: At the first level (single-subject), the emotional-faces/places task was modeled using a boxcar function with place blocks serving as the implicit baseline^63^. Block onsets were modeled as nuisance variates using two additional event-related predictors. All predictors were convolved with a canonical HRF. Prior research in relatively large samples has failed to uncover alcohol-induced changes in EAc blood flow, mitigating concerns about gross hemodynamic differences^64^. Additional nuisance variates included motion and physiological noise estimates. To attenuate physiological noise, white matter (WM) and cerebrospinal fluid (CSF) time-series were identified by thresholding the tissue prior images distributed with FSL. The EPI time-series was orthogonalized with respect to the first 3 right eigenvectors of the data covariance matrix from the WM and CSF compartments^65^.

Reactivity to emotional faces (i.e., the main effect of Stimulus: Emotional Faces vs. Places) was assessed using a voxelwise one-sample *t* test controlling for potential nuisance variance in mean-centered age and sex. The impact of alcohol administration was assessed using a voxelwise two-sample *t* test controlling for mean-centered age and sex, equivalent to testing the Stimulus (Emotional Faces vs. Places) × Treatment (Alcohol vs. Placebo) interaction.

#### Hypothesis Testing Strategy

The major aim of the study was to test the hypothesized dampening effects of acute alcohol administration on EAc reactivity to emotional faces.

EAc Region-of-Interest (ROI) Analyses: The Stimulus × Treatment interaction was rigorously thresholded at *p* < 0.05 familywise error (FWE) corrected for the extent of the EAc ROI, as in prior work by our group^66^. The EAc ROI encompassed the amygdala, substantia innominata/sublenticular extended amygdala (SI/SLEA), and BST bilaterally^40,67^. Consistent with recent recommendations^4,6^, the ROI was created using the Mai and Harvard-Oxford atlases^68–72^ and included the probabilistic BST ROI developed by Theiss and colleagues (*p* > 0%)^39^ and the Harvard-Oxford probabilistic amygdala. Using this as a starting point, voxels in the region of the SI/SLEA was manually added in the coronal plane of the 1-mm MNI152 template, working from rostral to caudal, and confirmed in the other planes. At intermediate levels of the amygdala’s rostral-caudal axis, where the BST was no longer visible, the SI/SLEA was limited to voxels dorsal to the amygdala and ventral to the putamen and pallidum. SI/SLEA voxels were included until the head of the hippocampus was clearly visible. Voxels in neighboring regions of the accumbens, caudate, putamen, pallidum, thalamus, and ventricles (Harvard-Oxford atlas, *p* > 50%) were excluded using a Boolean ‘NOT.’ The resulting bilateral EAc ROI was decimated to 2-mm^3^ (Supplementary Figure S2; total: 1,205 voxels; 9,640 mm^3^). Significant clusters (*p* < 0.05, whole-brain FWE corrected) outside the EAc are reported on an exploratory basis for voxelwise analyses of the Condition (Emotional Faces vs. Places) and Stimulus × Treatment effects.

Unbiased Comparison of the BST and Ce: In order to test the whether the BST and the Ce differ in their sensitivity to the hypothesized dampening effects of alcohol in an unbiased manner, we extracted and averaged standardized contrast coefficients using anatomically defined, *a priori* ROIs^39,40^ (Supplementary Figure S3). A general linear model was used to compare the impact of Treatment and Hemisphere on regional reactivity to emotional faces. Significant interactions were decomposed using simple effects. The Group effect is reported using the Welch-Satterthwaite correction (*F*_*W-S*_). A power analysis revealed that a minimum of 44 subjects is required to achieve 95% power to detect a Stimulus × Treatment interaction with a between-subjects design (as in the present study) and an estimated effect size of Cohen’s *d* = 1.13 (reported in ref.^25^) at *p* < 0.05, uncorrected.

## Results

### Behavior

On average, subjects were highly accurate at performing the simple discrimination tasks (*M* = 86.8%, *SD* = 7.9). Nevertheless, performance was ~8% lower in the alcohol (*M* = 83.2%, *SD* = 8.2) compared to the placebo group (*M* = 91.1%, *SD* = 4.9; *F*_*W-S*_(1,47) = 15.98, *p* < 0.001), consistent with prior work^73^. Subjects were ~4% more accurate when performing the places (*M* = 88.8%, *SD* = 8.8) compared to the faces discrimination (*M* = 84.4%, *SD* = 8.4; *F*(48) = 22.37, *p* < 0.001), but the Group × Condition interaction was not reliable (*F*(1,47) = 0.24, *p* = 0.63). As noted below, control analyses indicated that these modest differences in performance were not the primary determinant of alcohol-related differences in neural reactivity.

#### The Dorsal Amygdala is Sensitive to Emotional Faces

**Figure 2:**
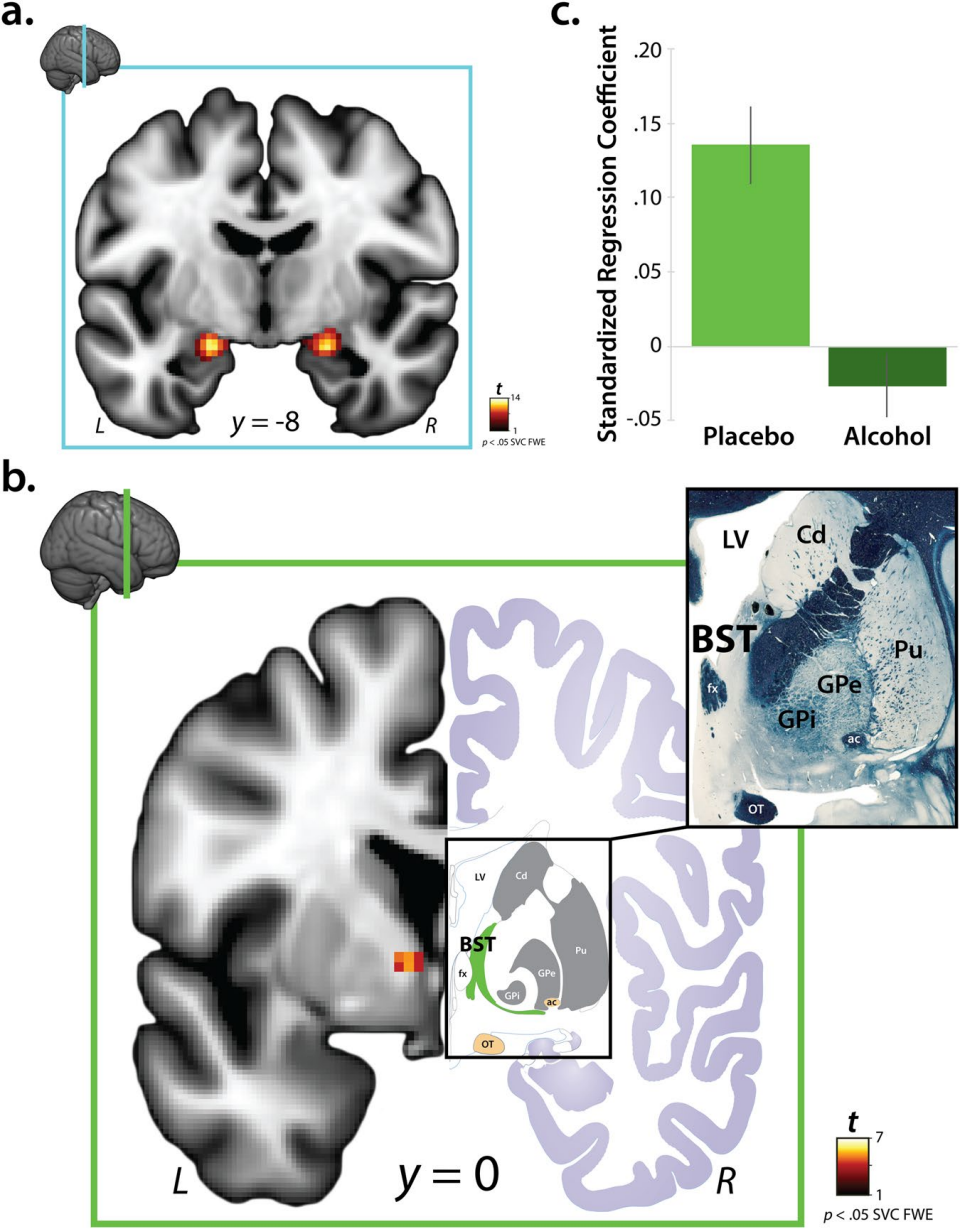
The impact of acute alcohol administration on reactivity to emotional faces in the central extended amygdala. (**a**) Consistent with prior work, voxelwise regression analyses revealed significant activation to emotional faces in the dorsal amygdala (*p* < 0.05, FWE corrected for the volume of the anatomically defined EAc region-of-interest; total volume: 1,205 voxels; 9,640 mm^3^). Inset indicates the location of the coronal slice. Significant clusters within the EAc ROI (Supplementary Figure S2) are depicted here. For additional results, see Supplementary Figures S4 and S5 and Supplementary Tables S1 and S2. (**b**) Voxelwise analyses revealed a significant reduction in reactivity to emotional faces in the region of the left BST in the alcohol compared to the placebo group (same threshold; equivalent to testing the Stimulus × Treatment interaction). The left half of the panel depicts the BST cluster. The right half depicts the BST (*green*) in the corresponding section of the human brain atlas^71^. Note the similar appearance of several key landmarks, including the fornix and lateral ventricle (*white*), as well as the optic tract and anterior commissure (*gold*). Upper left inset indicates the location of the coronal slice. Upper right inset depicts the myeloarchitecture (Weigert fiber stain) of this region in the atlas. The left BST was the only significant cluster in EAc-focused or whole-brain analyses. For additional results, see Supplementary Figure S6 and Supplementary Table S3. (**c**) For illustrative purposes, barplot depicts mean standardized regression coefficients extracted from the peak voxel in the BST cluster for the alcohol (*light green*) and placebo (*dark green*) groups. Hypothesis testing was performed on a voxelwise basis (corrected for multiple comparisons). Error bars indicate the standard error of the mean. Portions of this figure were adapted with permission from ref.^71^. Abbreviations—ac, anterior commissure; BST, bed nucleus of the stria terminalis; Cd, caudate; EAc, central division of the extended amygdala; FWE, family-wise error; fx, fornix; GPe, external globus pallidus; GPi, internal globus pallidus; L, left hemisphere; LV, lateral ventricle; OT, optic tract; Pu, putamen; R, right hemisphere; SVC, small volume correction.

Within the EAc, emotional faces were associated with significant activation of the dorsal amygdala, bilaterally (*p* < 0.05, FWE-corrected; Left: *t* = 12.59, volume = 1,032 mm^3^; *x* = −20, *y* = −10, *z* = −14; Right: *t* = 12.22, volume = 1,368 mm^3^; *x* = 22, *y* = −8, *z* = −16; Fig. 2a and Supplementary Table S1), consistent with prior work^10^. As shown in Supplementary Figure S4, the amygdala cluster overlapped the anatomically defined Ce ROI, with the left and right peaks lying in the dorsocaudal region where the Ce, medial, and basomedial nuclei abut.

On an exploratory basis, we also computed a series of whole-brain analyses. Results indicated that the dorsal amygdala and fusiform cortex (‘fusiform face area’) were significantly more sensitive to emotional faces, whereas the parahippocampal cortex (‘parahippocampal place area’) was significantly more sensitive to places, as expected^74,75^ (*p* < 0.05, FWE-corrected; Supplementary Figure S5 and Supplementary Table S2).

#### Alcohol Dampens BST Reactivity

Within the EAc, acute alcohol administration was associated with a significant reduction in left BST reactivity to emotional faces (Stimulus × Treatment: *p* < 0.05, FWE-corrected; *t* = 5.46, volume = 104 mm^3^; *x* = −8, *y* = −2, *z* = 0; Fig. 2b–c and Supplementary Table S3). As shown in Supplementary Figure S6, the left BST cluster overlapped the anatomically defined BST ROI. The Stimulus × Treatment interaction was not significant in the amygdala at this threshold. Exploratory whole-brain analyses revealed no additional clusters. Control analyses performed using a performance-matched sub-sample (*n* = 15/group) yielded similar results, suggesting that the dampening effects of alcohol on BST reactivity are not due to group differences in performance.

#### Alcohol Exerts Similar Effects in the Ce and the BST

**Figure 3:**
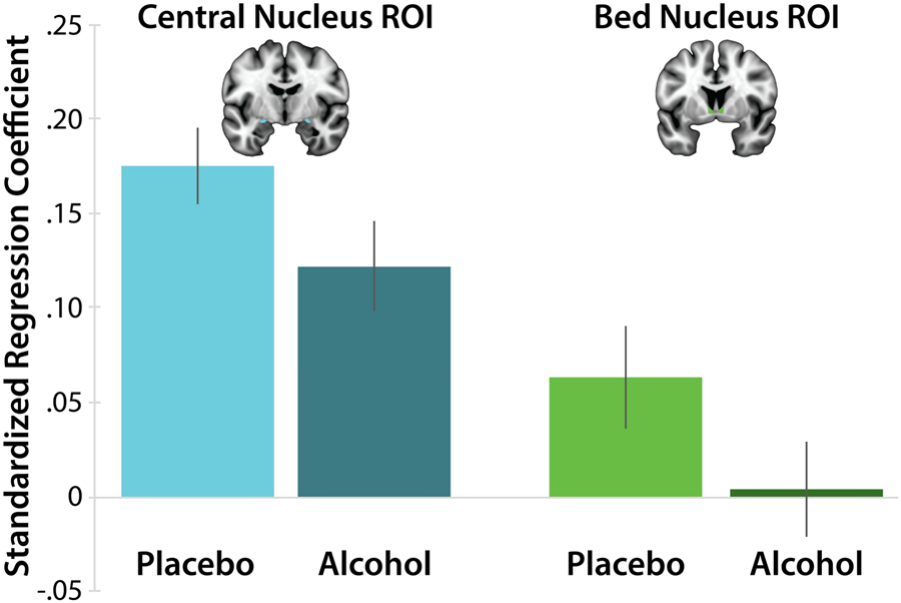
The impact of acute alcohol administration on the two major divisions of the EAc. Barplot depicts mean regression coefficients associated with the emotional-faces/places task for the anatomically defined Ce and BST ROIs for each group. The Ce was significantly more reactive to emotional faces, relative to the BST (*p* < 0.001). On average, subjects randomly assigned to the alcohol group showed significantly less reactivity to emotional faces, relative to those in the placebo group (*p* = 0.05; equivalent to testing the Stimulus × Treatment interaction). The Treatment × Region interaction was not significant (*p* = 0.88), suggesting that the Ce and BST are similarly sensitive to acute alcohol dampening. Error bars indicate the standard error of the mean. Abbreviations—EAc, central extended amygdala; ROI, region of interest.

To assess potential regional differences in EAc activation in an unbiased manner^76^, standardized contrast coefficients (i.e., emotional faces vs. places) were extracted from the left and right BST and Ce using anatomically defined, *a priori* ROIs, as shown in the upper portion of Fig. 3 (Ce: *cyan*; BST: *green*). A mixed-model GLM was then used to compare the impact of Treatment and Hemisphere on regional reactivity to emotional faces. Analyses revealed greater activation to faces in the Ce compared to the BST (Region: *F*(1,47) = 32.99, *p* < 0.001), consistent with recent high-resolution imaging research^77^. Analyses also revealed a significant alcohol-dampening effect across regions (Group: *F*_*W-S*_(1,47) = 3.93, *p* = 0.05). Other omnibus effects were not significant (*p*s > 0.15). Control analyses performed using a performance-matched sub-sample yielded similar results. Collectively, these observations indicate that alcohol acutely dampens EAc reactivity to emotional faces, it does so similarly in the BST and Ce, and these effects are not an artifact of group differences in task engagement.

## Discussion

Recent epidemiological work indicates that the United States is facing a growing alcohol use crisis^78^, yet the neural circuitry most relevant to human alcohol consumption has remained unclear. Leveraging a placebo-controlled, randomized-groups design, our voxelwise results demonstrate for the first time that alcohol acutely dampens BST reactivity to emotional faces (Fig. 2). Analyses performed using unbiased, anatomically defined ROIs revealed similar patterns of reduced reactivity in the BST and the Ce (Fig. 3). Control analyses indicated that these results were not an artifact of group differences in performance. Collectively, these findings indicate that acute alcohol intoxication dampens reactivity to emotional faces and it does so similarly across the major divisions of the EAc.

The present findings are broadly consistent with models of alcohol drinking derived from preclinical research in mice and rats^14–17^. This work strongly implicates both divisions of the EAc in the anxiety-reducing consequences of alcohol^19,23^. Alcohol robustly engages the BST and the Ce, as indexed by elevated expression of the immediate early gene c-*fos*^20^. Acute alcohol consumption is associated with reduced behavioral signs of anxiety and increased c-*fos* induction in the BST and Ce^21^. While the molecular consequences of alcohol are complex, alcohol acutely inhibits excitatory (i.e., glutamatergic) neurotransmission across the EAc and increases inhibitory (i.e., GABA) neurotransmission in the Ce^79–81^. Other work indicates that EAc microcircuits play a critical role in excessive drinking^82,83^, consistent with evidence implicating the EAc in withdrawal-induced signs of anxiety and stress-induced substance use^14^. Although these observations highlight the importance of specific cell types and circuits within and between the Ce and BST for alcohol consumption in rodents, the relevance of these discoveries to human drinking and disease has remained unclear. The present results, which underscore the similar consequences of acute alcohol consumption across the EAc, increase our confidence that the pathways identified in rodent models are broadly conserved across species and can guide the development of improved treatments^84^. The development of integrative animal models that combine focal perturbations of the EAc with the same kinds of paradigms and imaging techniques routinely used in human studies would allow a more complete and detailed synthesis of these distinct bodies of research^4,85^.

### Future Challenges

Although the present study affords new insights into the acute impact of alcohol on the human brain, several limitations and challenges merit comment. First, while single-blind designs are routinely used in acute alcohol challenge studies^86^, use of a double-blind design would eliminate potential experimenter-expectancy biases. Second, the present study used static images of fearful (75%) and emotionally neutral (25%) faces to probe EAc reactivity. Although fearful faces do not elicit robust signs of fear or anxiety^10^ (e.g. potentiation of the startle reflex) and are less ecologically valid than dynamic expressions of emotion^87^, they are widely used in neuroimaging research, rated as more threatening and arousing than neutral or happy faces, and associated with increased behavioral caution^88–91^. Fearful faces also promote vigilance; the mere presentation of fearful faces produces persistent increases in visual sensitivity, boosts the resolution of visual processing, and enhances the efficiency of attentional search^10^. Vigilance is thought to be mediated by circuits emanating from the EAc^11^ and, once elicited, increases the likelihood of experiencing more extreme or pervasive states of distress^10,92^. Notably, recent neuroimaging research indicates that individuals with elevated amygdala reactivity to fearful faces are more likely to abuse alcohol in the future, during and following exposure to negative life events (e.g. significant academic, financial, health, or relationship problems)^42,43^. The present results reinforce the possibility that this prospective association reflects attempts to downregulate, normalize, or self-medicate neural circuits centered on the EAc. Testing this hypothesis is an important challenge for future research. Assessing whether our conclusions generalize to more intense cues, such as threat-of-shock, represents another important avenue. The use of stimuli that elicit robust signs of anxiety (e.g. startle potentiation) would dovetail with work in rodent models, enhancing the likelihood of successful bi-directional translation^85,93^. Combined with more naturalistic measures of stress-induced drinking in the laboratory or field (e.g., using ecological momentary assessment), this approach might provide a means of stratifying at-risk populations or patients into the subset for whom negative reinforcement circuits are most relevant to intervention.

### Conclusions

Existing treatments for excessive alcohol consumption are far from curative^94,95^, highlighting the need for a deeper understanding of the underlying neural and motivational systems. The present results demonstrate that alcohol acutely dampens EAc reactivity in humans, providing an important source of validation for models of alcohol drinking derived from preclinical research in rodents. The use of a relatively large sample, placebo-controlled between-groups design, ecologically relevant dosing, fMRI-optimized task, best practices for the acquisition and processing of functional neuroimaging data, and unbiased ROI analytic approach enhances our confidence in the clinical and translational significance of these results. More broadly, these observations provide insights into some of the neural systems most relevant to the consumption of alcohol and the initial development of alcohol abuse in humans.

## Electronic supplementary material

Supplementary Information

## Data Availability

Key statistical maps and regions-of-interest are available at NeuroVault.org (http://neurovault.org/collections/4414/).
