# Supplementary material for: Acute alcohol administration dampens central extended amygdala reactivity
Source: Sci Rep. 2018 Nov 12;8:16702. doi: 10.1038/s41598-018-34987-3 (PMC6232084; doi:10.1038/s41598-018-34987-3)
Supplement: Supplementary file 1 — Supplementary Information [file 41598_2018_34987_MOESM1_ESM.docx]

Supplementary Method and Results to accompany—

*Acute alcohol administration dampens central extended amygdala reactivity*

Juyoen Hur^1^*

Claire M. Kaplan^1^*

Jason F. Smith^1^*

Daniel E. Bradford^4^

Andrew S. Fox^5,6^

John J. Curtin^4^

Alexander J. Shackman^1-3^

^1^Department of Psychology, ^2^Neuroscience and Cognitive Science Program, and ^3^Maryland Neuroimaging Center, University of Maryland, College Park, MD 20742 USA. ^4^Department of Psychology, University of Wisconsin—Madison, 1202 West Johnson Street, Madison, WI 53706 USA. ^5^Department of Psychology and ^6^California National Primate Research Center, University of California, Davis, CA 95616 USA

* contributed equally

**Address Correspondence to:**

Alexander J. Shackman ([shackman@umd.edu](mailto:shackman@umd.edu))

Biology-Psychology Building

University of Maryland

College Park MD 20742 USA


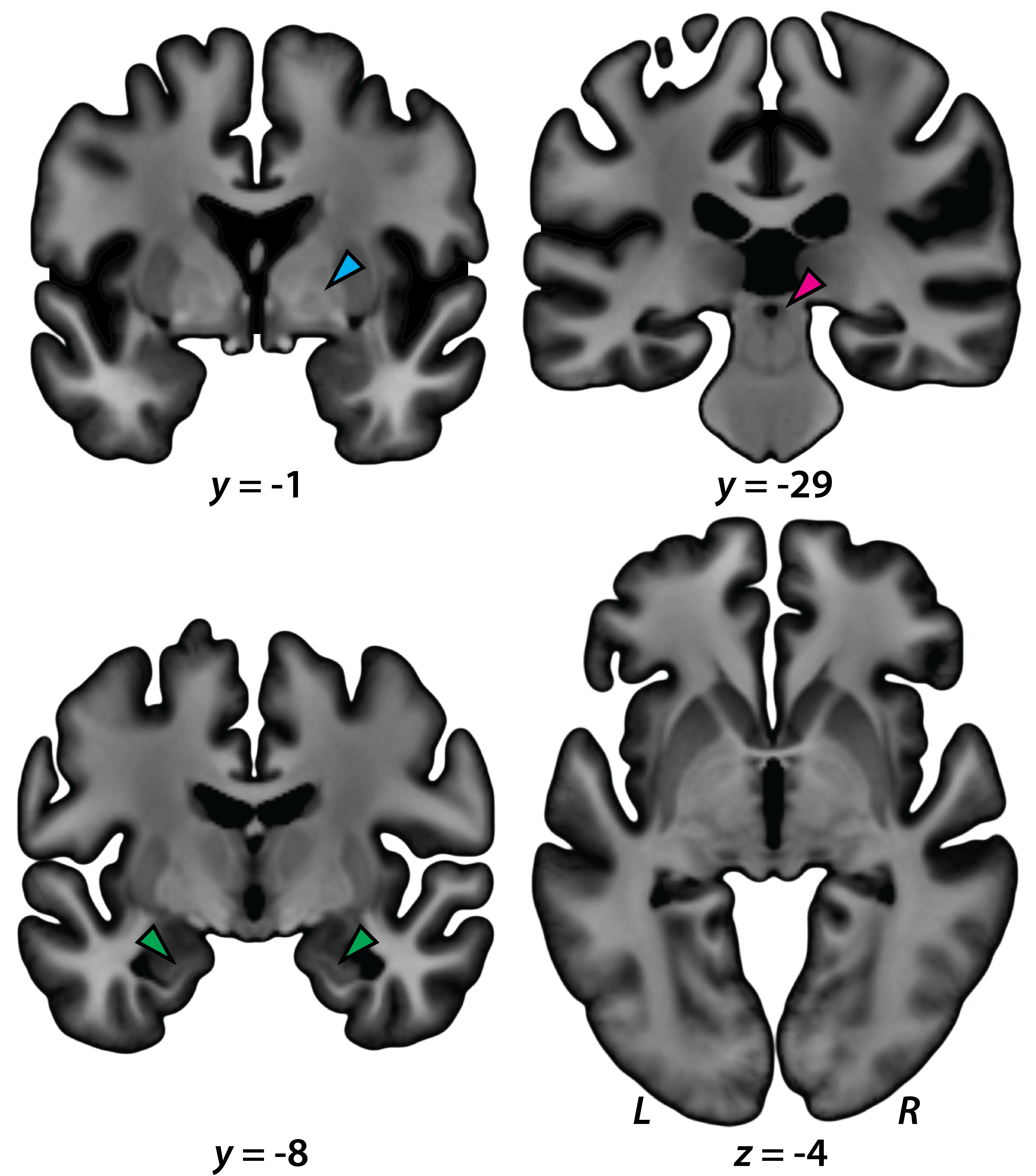


S**upplementary Figure S1.** ***Mean normalized T1 image***. Figure depicts representative slices from the average of the 49 diffeomorphically normalized T1-weighted images. Note the preservation of fine detail in the medial medullary lamina of the globus pallidus (*cyan* arrowhead), periaqueductal gray (*magenta* arrowhead), and alveus (*green* arrowheads).

***
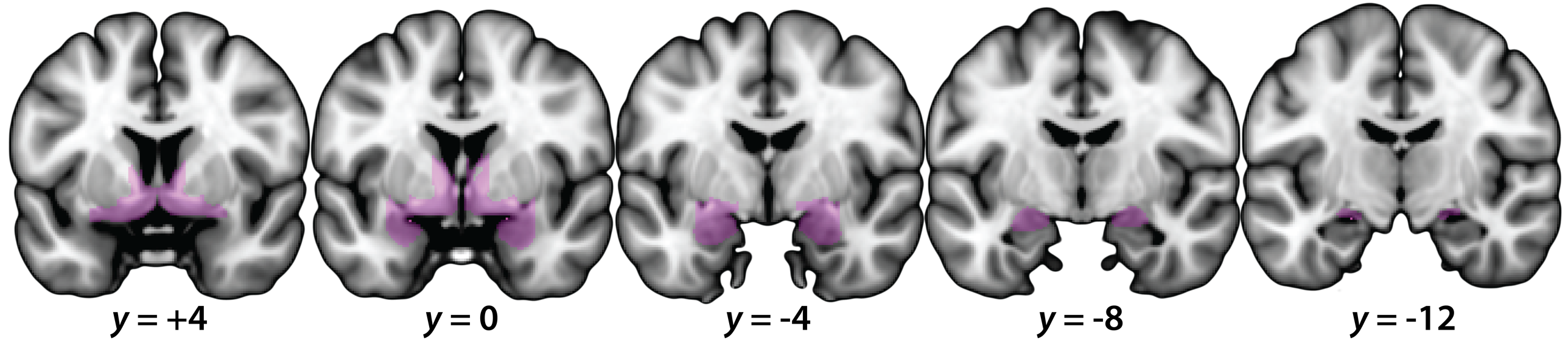
***

**Supplementary Figure S2. EAc ROI.** The EAc ROI (*purple*) encompassed the amygdala, substantia innominata/sublenticular extended amygdala (SI/SLEA), and BST bilaterally [^1^](#_ENREF_1). Consistent with recent recommendations [^2^](#_ENREF_2)^,^[^3^](#_ENREF_3), the ROI was created using a combination of the Mai and Harvard-Oxford atlases [^4-8^](#_ENREF_4) and included the probabilistic BST ROI developed by Theiss and colleagues (*p*>0%) [^9^](#_ENREF_9) and the Harvard-Oxford probabilistic amygdala (*p*>50%). Using this as a starting point, voxels in the region of the SI/SLEA was manually added in the coronal plane of the 1-mm MNI152 template, working from rostral to caudal, and confirmed in the other planes. At intermediate levels of the amygdala’s rostral-caudal axis, where the BST was no longer visible, the SI/SLEA was limited to voxels dorsal to the amygdala and ventral to the putamen and pallidum. SI/SLEA voxels were included until the head of the hippocampus was clearly visible. Voxels in neighboring regions of the accumbens, caudate, putamen, pallidum, thalamus, and ventricles (Harvard-Oxford atlas, *p*>50%) were excluded using a Boolean ‘NOT.’ The resulting bilateral ROI was decimated to 2-mm^3^ (total: 1,205 voxels; 9,640 mm^3^). For illustrative purposes, the 1-mm ROI is shown.

**
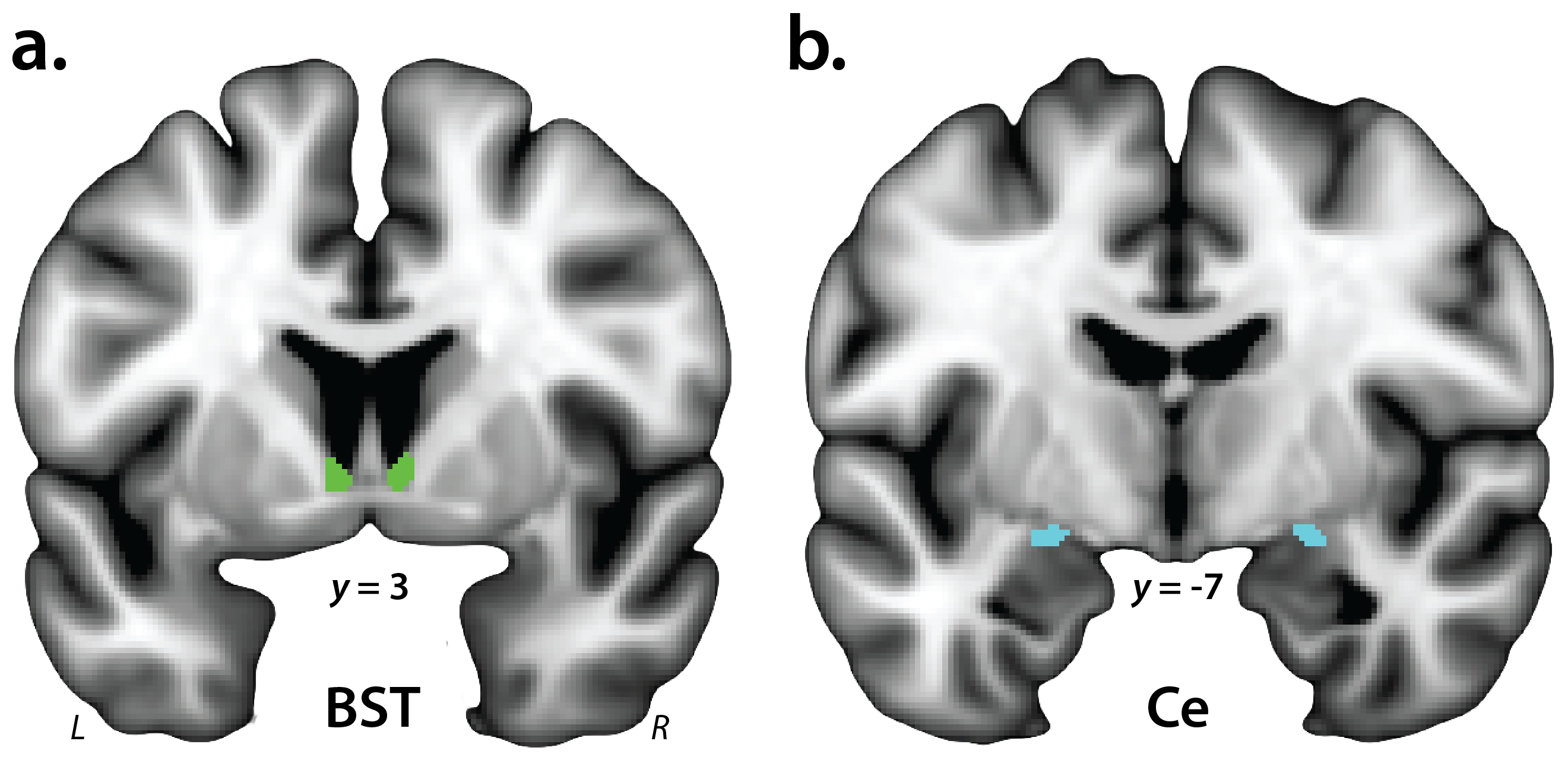
Supplementary Figure S3. BST and Ce ROIs.** ***a. BST.*** The derivation of the probabilistic BST ROI (*green*) is detailed in [^9^](#_ENREF_9) and was thresholded at 25%. The seed mostly encompasses the supra-commissural BST, given the difficulty of reliably discriminating the borders of regions below the anterior commissure on the basis of T1-weighted images [cf. ^10^](#_ENREF_10). ***b. Ce.*** The derivation of the Ce ROI (*cyan*) is described in more detail in Tillman et al. (2018). For illustrative purposes, 1-mm ROIs are shown. Analyses employed ROIs decimated to the 2-mm resolution of the EPI data. Single-subject data were visually inspected to ensure that the ROIs were correctly aligned to the spatially normalized T1 images. Abbreviations—BST, bed nucleus of the stria terminalis; Ce, central nucleus of the amygdala; L, left hemisphere; R, right hemisphere.


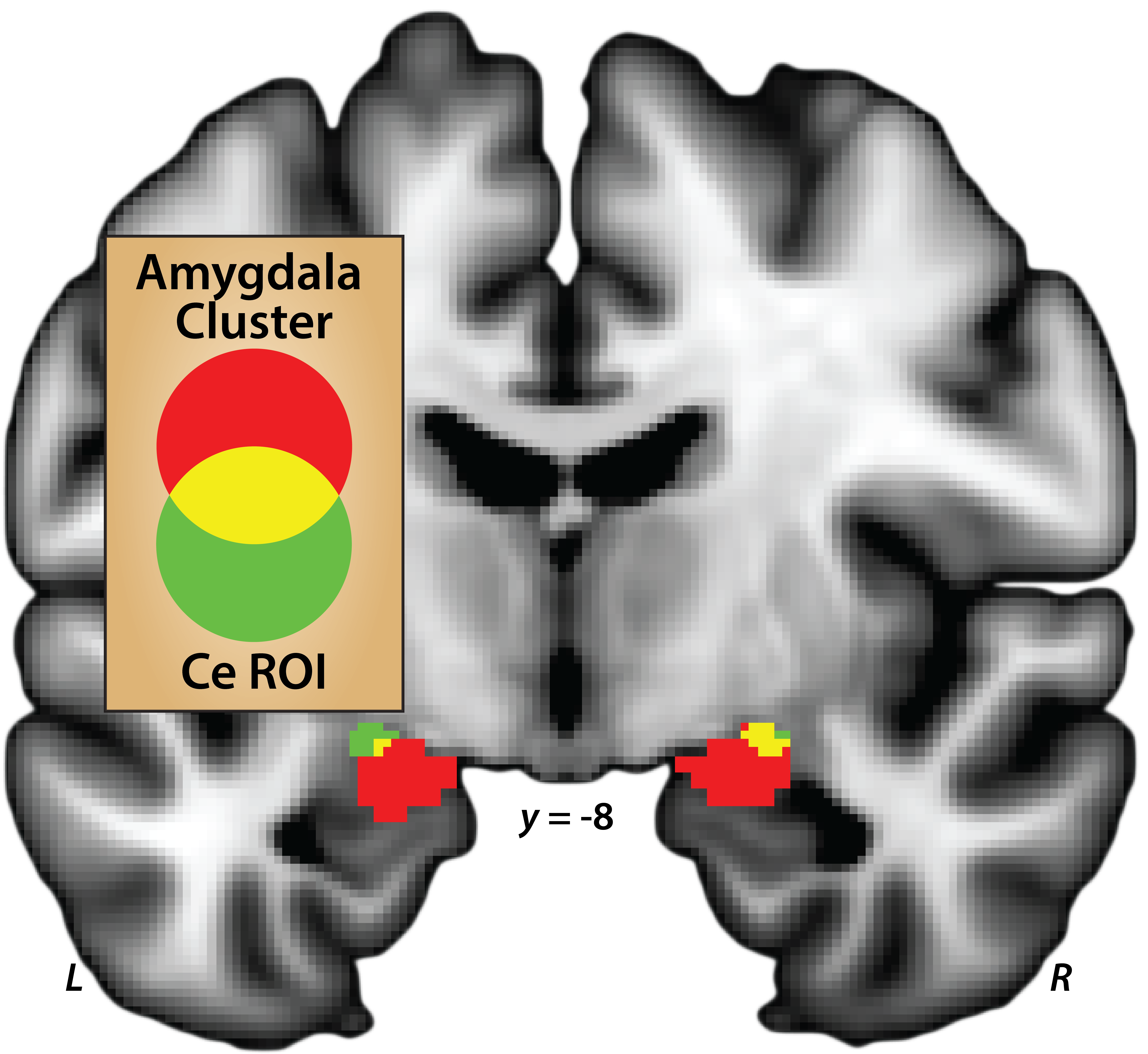
**Supplementary Figure S4. *The amygdala cluster identified in voxelwise analyses overlaps the anatomically defined Ce ROI.*** While the cluster included numerous amygdala nuclei, the left (*x*=-20, *y*=-10, *z*=-14) and right (*x*=22, *y*=-8, *z*=-16) peaks lie in the dorsocaudal region where the more dorsal Ce and more ventral basomedial nuclei abut. The derivation of the Ce seed (*green*) is described in more detail in Tillman et al. (2018). Abbreviations—Ce, central nucleus of the amygdala; L, left hemisphere; R, right hemisphere; ROI, region of interest.

**Supplementary Table S1*. Descriptive statistics for clusters identified by the emotional faces vs. places contrast using p<.05, small-volume corrected^a^***

|  | | **mm^3^** | ***t*** | ***x*** | ***y*** | ***z*** |
| --- | --- | --- | --- | --- | --- | --- |
|  | ***Faces > Places*** |  |  |  |  |  |
|  | R Frontal | 13,824 |  |  |  |  |
|  | R Frontal Pole^b^ |  | 4.67 | 50 | 38 | 28 |
|  | R Inferior Frontal Gyrus, pars opercularis^b^ |  | 8.92 | 54 | 12 | 22 |
|  | R Precentral Gyrus^b^ |  | 6.53 | 44 | -2 | 40 |
|  | L Insular Cortex^b^ | 696 | 6.15 | -34 | 24 | 0 |
|  | R Frontal Operculum Cortex^b^ | 768 | 6.65 | 38 | 24 | 0 |
|  | R Superior Frontal Gyrus^b^ | 344 | 5.05 | 6 | 18 | 56 |
|  | L Inferior Frontal Gyrus^b^ | 328 | 4.93 | -44 | 16 | 26 |
|  | L Temporal/Amygdala | 2,480 |  |  |  |  |
|  | L Temporal Pole^b^ |  | 5.48 | -34 | 4 | -28 |
|  | L Parahippocampal Gyrus, anterior^b^ |  | 6.05 | -32 | 0 | -32 |
|  | L Dorsal Amygdala^c^ |  | 12.59 | -20 | -10 | -14 |
|  | R Posterior Temporal/Amygdala | 2,832 |  |  |  |  |
|  | R Temporal Fusiform Cortex, anterior^b^ |  | 5.41 | 32 | -2 | -34 |
|  | R Inferior Temporal Gyrus, anterior^b^ |  | 5.94 | 40 | -2 | -40 |
|  | R Dorsal Amygdala^c^ |  | 12.22 | 22 | -8 | -16 |
|  | R Thalamus^b^ | 88 | 4.64 | 6 | -4 | 0 |
|  | L Postcentral Gyrus^b^ | 1,208 | 7.39 | -48 | -18 | 48 |
|  | R Temporal-Occipital | 14,696 |  |  |  |  |
|  | R Middle Temporal Gyrus, posterior^b^ |  | 6.32 | 50 | -26 | -4 |
|  | R Middle Temporal Gyrus, temporooccipital part^b^ |  | 10.29 | 54 | -60 | 10 |
|  | R Lateral Occipital Cortex, inferior^b^ |  | 11.51 | 54 | -68 | 6 |
|  | R Supramarginal Gyrus, anterior^b^ | 352 | 4.78 | 54 | -34 | 52 |
|  | R Occipital-Temporal | 3,184 |  |  |  |  |
|  | R Inferior Temporal Gyrus^b^ |  | 7.75 | 46 | -40 | -18 |
|  | R Temporal Occipital Fusiform Cortex^b^ |  | 9.81 | 42 | -48 | -20 |
|  | L Temporal Occipital Fusiform Cortex^b^ | 2,416 | 10.22 | -40 | -50 | -18 |
|  | L Occipital-Temporal | 5,040 |  |  |  |  |
|  | L Middle Temporal Gyrus, temporooccipital part^b^ |  | 6.08 | -58 | -52 | 10 |
|  | L Lateral Occipital Cortex, inferior^b^ |  | 7.19 | -52 | -68 | 8 |
|  | L Intracalcarine Cortex^b^ | 128 | 4.67 | -8 | -88 | 10 |
|  | ***Places > Faces*** |  |  |  |  |  |
|  | Bilateral Frontal | 6,808 |  |  |  |  |
|  | L Frontal Pole^b^ |  | 4.85 | -2 | 62 | 2 |
|  | R Frontal Pole^b^ |  | 4.78 | 6 | 58 | 2 |
|  | L Cingulate Gyrus, anterior^b^ |  | 6.03 | -4 | 40 | -4 |
|  | R Paracingulate Gyrus^b^ |  | 6.05 | 6 | 40 | -6 |
|  | R Cingulate Gyrus, anterior^b^ |  | 5.83 | 4 | 38 | 18 |
|  | L Paracingulate Gyrus^b^ |  | 5.58 | -10 | 38 | -6 |
|  | L Frontal Pole^b^ | 120 | 5.01 | -22 | 50 | 28 |
|  | L Frontal | 2,816 |  |  |  |  |
|  | L Frontal Pole^b^ |  | 5.38 | -18 | 40 | 40 |
|  | L Middle Frontal Gyrus^b^ |  | 4.67 | -28 | 26 | 40 |
|  | L Superior Frontal Gyrus^b^ |  | 6.69 | -18 | 26 | 48 |
|  | L Frontal Pole^b^ | 384 | 5.49 | -34 | 38 | -10 |
|  | R Frontal Pole^b^ | 128 | 4.67 | 32 | 38 | -10 |
|  | R Middle Frontal Gyrus^b^ | 256 | 4.87 | 28 | 26 | 36 |
|  | R Cingulate Gyrus, anterior^b^ | 248 | 4.47 | 2 | 6 | 32 |
|  | L Insular Cortex^b^ | 304 | 5.34 | -44 | 4 | -6 |
|  | R Temporal-Parietal | 4,248 |  |  |  |  |
|  | R Precentral Gyrus^b^ |  | 4.71 | 62 | 0 | 10 |
|  | R Superior Temporal Gyrus, posterior^b^ |  | 5.78 | 66 | -20 | 4 |
|  | R Planum Temporale^b^ |  | 6.14 | 64 | -26 | 12 |
|  | R Parietal Operculum Cortex^b^ |  | 6.58 | 50 | -28 | 24 |
|  | L Temporal | 880 |  |  |  |  |
|  | L Middle Temporal Gyrus, anterior^b^ |  | 5.31 | -62 | -2 | -12 |
|  | L Superior Temporal Gyrus, anterior^b^ |  | 4.45 | -60 | -6 | -6 |
|  | L Middle Temporal Gyrus, posterior^b^ |  | 5.23 | -66 | -18 | -10 |
|  | L Temporal | 728 |  |  |  |  |
|  | L Superior Temporal Gyrus, anterior^b^ |  | 5.40 | -62 | -4 | 4 |
|  | L Central Opercular Cortex^b^ |  | 4.59 | -56 | -6 | 10 |
|  | R Inferior Visual Cortex | 188,488 |  |  |  |  |
|  | R Parahippocampal Gyrus, posterior^b^ |  | 10.09 | 20 | -24 | -20 |
|  | R Thalamus^b^ |  | 8.31 | 24 | -32 | 4 |
|  | L Thalamus^b^ |  | 9.55 | -20 | -32 | -2 |
|  | L Parahippocampal Gyrus, anterior^b^ |  | 15.72 | -24 | -38 | -14 |
|  | R Lingual Gyrus^b^ |  | 20.56 | 24 | -40 | -12 |
|  | R Cingulate Gyrus, posterior^b^ |  | 11.06 | 10 | -46 | 4 |
|  | L Temporal Occipital Fusiform Cortex^b^ |  | 19.16 | -28 | -50 | -10 |
|  | L Lingual Gyrus^b^ |  | 12.50 | -16 | -52 | 2 |
|  | R Temporal Occipital Fusiform Cortex^b^ |  | 22.04 | 30 | -54 | -10 |
|  | R Precuneus Cortex^b^ |  | 16.79 | 18 | -58 | 12 |
|  | L Precuneus Cortex^b^ |  | 16.27 | -16 | -60 | 8 |
|  | L Occipital Fusiform Gyrus^b^ |  | 17.06 | -26 | -70 | -12 |
|  | R Occipital Fusiform Gyrus^b^ |  | 17.21 | 26 | -72 | -8 |
|  | L Lateral Occipital Cortex, superior ^b^ |  | 9.52 | -22 | -84 | 38 |
|  | R Lateral Occipital Cortex, superior^b^ |  | 14.29 | 40 | -86 | 12 |
|  | L Occipital Pole^b^ |  | 14.11 | -30 | -92 | 16 |
|  | R Occipital Pole^b^ |  | 13.53 | 18 | -98 | 0 |
|  | L Parietal Operculum Cortex^b^ | 336 | 4.96 | -60 | -28 | 16 |
|  | L Angular Gyrus^b^ | 384 | 5.38 | -50 | -56 | 30 |
|  | L Middle Temporal Gyrus, temporooccipital^b^ | 1,912 | 6.94 | -58 | -58 | -8 |

**^a^** The faces-vs.-places contrast was thresholded at *p*<.05 FWE-corrected for the extent of the EAc ROI (1,205 voxels; 9,640 mm^3^). For transparency, all clusters greater than or equal to 80 mm^3^ (10 native EPI voxels) are reported. ^b^ Lies outside the *a priori* EAc ROI and is not significant. ^c^ Lies inside the *a priori* EAc ROI and is significant.


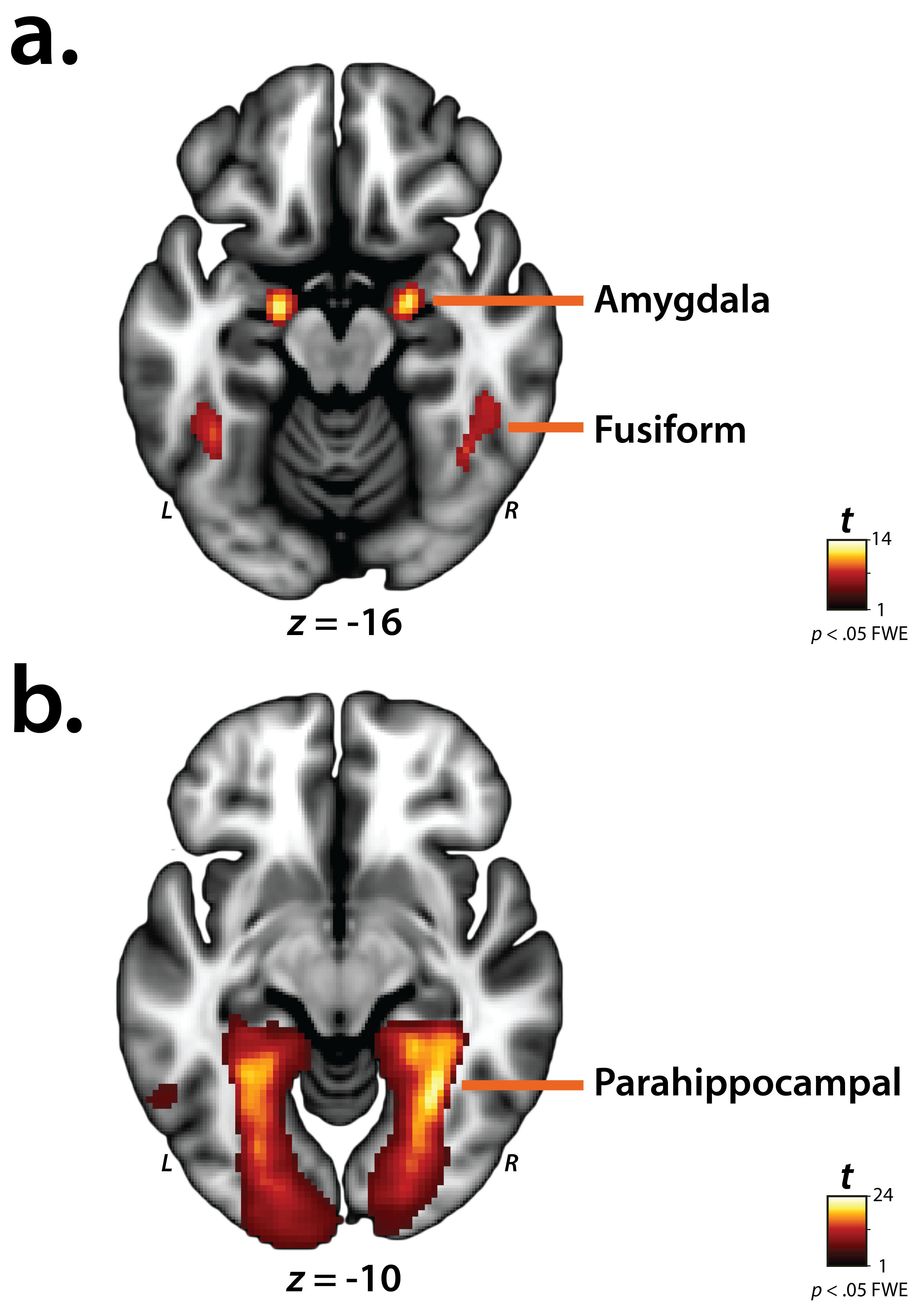
**Supplementary Figure S5. *Regions identified by a whole-brain, voxelwise regression analysis (p < .05, whole-brain FWE corrected). A.*** The amygdala and fusiform cortex show significantly greater activation to emotional faces than places***. B.*** The parahippocampal cortex shows significantly greater activation to places than emotional faces***.*** Abbreviations—L, left hemisphere; R, right hemisphere.

**Supplementary Table S2*. Descriptive statistics for clusters identified by the emotional faces vs. places contrast using p<.05, whole-brain corrected^a^***

|  | | **mm^3^** | ***T*** | ***x*** | ***y*** | ***z*** |
| --- | --- | --- | --- | --- | --- | --- |
|  | ***Faces > Houses*** |  |  |  |  |  |
|  | R Frontal Operculum Cortex | 80 | 6.65 | 38 | 24 | 0 |
|  | R Inferior Frontal Gyrus, pars opercularis | 3,640 | 8.92 | 54 | 12 | 22 |
|  | R Precentral Gyrus | 224 | 6.53 | 44 | -2 | 40 |
|  | L Amygdala | 800 | 12.59 | -20 | -10 | -14 |
|  | R Amygdala | 920 | 12.22 | 22 | -8 | -16 |
|  | L Precentral | 360 |  |  |  |  |
|  | L Postcentral Gyrus |  | 7.39 | -48 | -18 | 48 |
|  | L Precentral Gyrus |  | 6.09 | -36 | -22 | 50 |
|  | R Temporal-Occipital | 5,936 |  |  |  |  |
|  | R Middle Temporal Gyrus, posterior |  | 6.32 | 50 | -26 | -4 |
|  | R Middle Temporal Gyrus, temporooccipital part |  | 7.69 | 54 | -40 | 2 |
|  | R Lateral Occipital Cortex, inferior |  | 11.51 | 54 | -68 | 6 |
|  | R Temporal-Occipital | 1,536 |  |  |  |  |
|  | R Inferior Temporal Gyrus, temporooccipital part |  | 7.75 | 46 | -40 | -18 |
|  | R Temporal Occipital Fusiform Cortex |  | 9.81 | 42 | -48 | -20 |
|  | L Temporal Occipital Fusiform Cortex | 1,144 | 10.22 | -40 | -50 | -18 |
|  | L Temporal-Occipital | 1,000 |  |  |  |  |
|  | L Middle Temporal Gyrus, temporooccipital part |  | 6.08 | -58 | -52 | 10 |
|  | L Lateral Occipital Cortex, inferior |  | 7.19 | -52 | -68 | 8 |
|  | ***Places > Faces*** |  |  |  |  |  |
|  | L Superior Frontal Gyrus (C7) | 184 | 6.69 | -18 | 26 | 48 |
|  | R Inferior Visual Cortex | 55,360 |  |  |  |  |
|  | R Hippocampus |  | 8.77 | 30 | -20 | -22 |
|  | R Parahippocampal Gyrus |  | 10.09 | 20 | -24 | -20 |
|  | R Lingual Gyrus |  | 20.56 | 24 | -40 | -12 |
|  | R Temporal Occipital Fusiform Cortex |  | 22.04 | 30 | -54 | -10 |
|  | R Lateral Occipital Cortex, superior |  | 15.54 | 36 | -88 | 16 |
|  | L Thalamus | 288 | 9.55 | -20 | -32 | -2 |
|  | R Thalamus | 240 | 8.31 | 24 | -32 | 4 |
|  | L Posterior Cingulate/Precuneus | 4,704 |  |  |  |  |
|  | L Cingulate Gyrus, posterior |  | 8.38 | -6 | -34 | 36 |
|  | L Precuneus Cortex |  | 7.97 | -8 | -42 | 44 |
|  | L Inferior Visual Cortex | 60,288 |  |  |  |  |
|  | L Parahippocampal Gyrus, posterior |  | 15.72 | -24 | -38 | -14 |
|  | L Temporal Occipital Fusiform Gyrus |  | 19.49 | -28 | -52 | -6 |
|  | L Cingulate Gyrus, posterior |  | 12.50 | -16 | -52 | 2 |
|  | L Precuneous Cortex |  | 16.27 | -16 | -60 | 8 |
|  | L Occipital Fusiform Gyrus |  | 17.06 | -26 | -70 | -12 |
|  | L Lateral Occipital Cortex, superior |  | 9.52 | -22 | -84 | 38 |
|  | L Occipital Pole |  | 14.11 | -30 | -92 | 16 |
|  | L Middle Temporal Gyrus | 424 | 6.94 | -58 | -58 | -8 |

^a^Clusters greater than or equal to 80 mm^3^ (10 native EPI voxels) are reported.


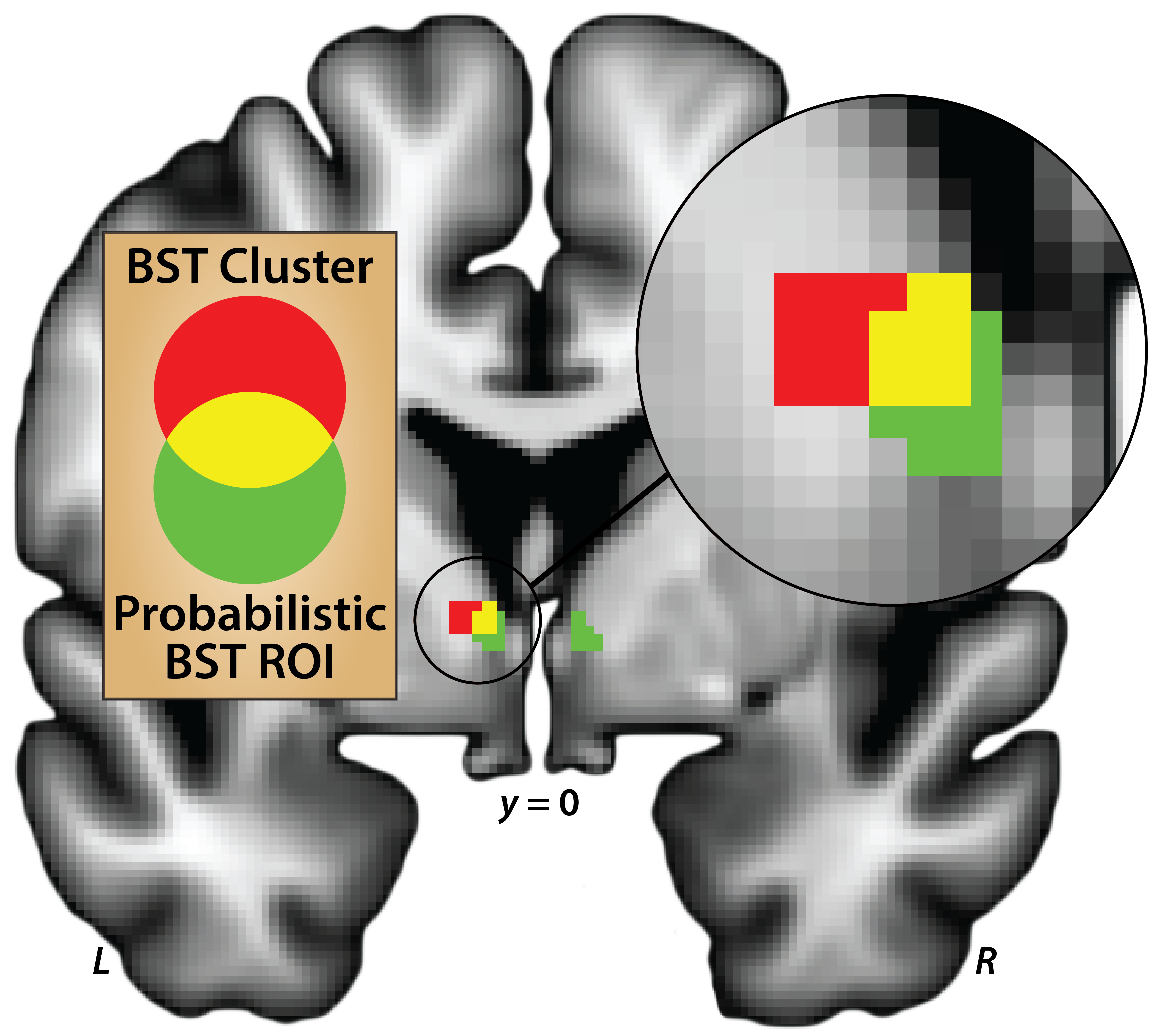
**Supplementary Figure S6. *The BST cluster identified in voxelwise analyses overlaps the anatomically defined BST ROI.*** The derivation of the probabilistic BST ROI (*green*) is detailed in [^9^](#_ENREF_9). The same pattern was evident using other available BST ROIs (not shown; [^11^](#_ENREF_11)^,^[^12^](#_ENREF_12). Abbreviations—L, left hemisphere; R, right hemisphere; ROI, region of interest.

**Supplementary Table S3*. Descriptive statistics for clusters identified by the Stimulus × Treatment contrast using p<.05, small-volume corrected^a^***

|  | | **mm^3^** | ***T*** | ***x*** | ***y*** | ***z*** |
| --- | --- | --- | --- | --- | --- | --- |
|  | ***Alcohol < Placebo: Faces minus Places*** |  |  |  |  |  |
|  | L Insula | 168 |  |  |  |  |
|  | L Frontal Operculum Cortex^b^ |  | 4.19 | -32 | 22 | 8 |
|  | L Insular Cortex^b^ |  | 5.46 | -34 | 20 | 2 |
|  | L BST^c^ | 104 | 5.46 | -8 | -2 | 0 |
|  | L Thalamus^b^ | 80 | 5.15 | -6 | -24 | 0 |
|  | ***Alcohol > Placebo: Faces minus Places*** |  |  |  |  |  |
|  | R Temporal Occipital Fusiform Cortex^b^ | 360 | 5.94 | 26 | -58 | -12 |
|  | R Lateral Occipital Cortex, superior^b^ | 200 | 5.10 | 32 | -80 | 12 |

^a^The Stimulus × Treatment interaction contrast was thresholded at *p*<.05 FWE-corrected for the extent of the EAc ROI (1,205 voxels; 9,640 mm^3^). For transparency, all clusters greater than or equal to 80 mm^3^ (10 native EPI voxels) are reported. ^b^Lies outside the *a priori* EAc ROI and is not significant. ^c^Lies inside the *a priori* EAc ROI and is significant.

**SUPPLEMENTARY REFERENCES**

1 Yilmazer-Hanke, D. M. in *The human nervous system* (eds J. K. Mai & G. Paxinos) 759–834 (Academic Press, 2012).

2 Shackman, A. J. & Fox, A. S. Contributions of the central extended amygdala to fear and anxiety. *J. Neurosci.* **36**, 8050-8063 (2016).

3 Fox, A. S. & Shackman, A. J. The central extended amygdala in fear and anxiety: Closing the gap between mechanistic and neuroimaging research. *Neuroscience letters* (in press).

4 Desikan, R. S. *et al.* An automated labeling system for subdividing the human cerebral cortex on MRI scans into gyral based regions of interest. *Neuroimage* **31**, 968-980 (2006).

5 Frazier, J. A. *et al.* Structural brain magnetic resonance imaging of limbic and thalamic volumes in pediatric bipolar disorder. *American Journal of Psychiatry* **162**, 1256-1265 (2005).

6 Goldstein, J. M. *et al.* Hypothalamic abnormalities in schizophrenia: sex effects and genetic vulnerability. *Biological psychiatry* **61**, 935-945 (2007).

7 Mai, J. K., Majtanik, M. & Paxinos, G. *Atlas of the human brain*. (Academic Press, 2015).

8 Makris, N. *et al.* Decreased volume of left and total anterior insular lobule in schizophrenia. *Schizophrenia research* **83**, 155-171 (2006).

9 Theiss, J. D., Ridgewell, C., McHugo, M., Heckers, S. & Blackford, J. U. Manual segmentation of the human bed nucleus of the stria terminalis using 3T MRI. *Neuroimage* **146**, 288-292, doi:10.1016/j.neuroimage.2016.11.047 (2017).

10 Kruger, O., Shiozawa, T., Kreifelts, B., Scheffler, K. & Ethofer, T. Three distinct fiber pathways of the bed nucleus of the stria terminalis to the amygdala and prefrontal cortex. *Cortex* **66**, 60-68, doi:10.1016/j.cortex.2015.02.007 (2015).

11 Pauli, W. M., Nili, A. N. & Tyszka, J. M. A high-resolution probabilistic in vivo atlas of human subcortical brain nuclei. *bioRxiv* (2017).

12 Torrisi, S. *et al.* Resting state connectivity of the bed nucleus of the stria terminalis at ultra-high field. *Hum Brain Mapp* **36**, 4076-4088, doi:10.1002/hbm.22899 (2015).
